# Mouse models of B cell lymphomas on the basis of genetic features

**DOI:** 10.3389/fimmu.2026.1870989

**Published:** 2026-08-03

**Authors:** Min Yuan, James Q. Wang

**Affiliations:** 1 Department of Hematology, The Second Affiliated Hospital, Zhejiang University School of Medicine, Zhejiang University, Hangzhou, China; 2 Zhejiang University – University of Edinburgh Institute, Zhejiang University School of Medicine, Zhejiang University, Haining, China; 3 Deanery of Biomedical Sciences, Edinburgh Medical School, College of Medicine and Veterinary Medicine, The University of Edinburgh, Edinburgh, United Kingdom

**Keywords:** diffuse large B cell lymphoma, genetically engineered mouse models (GEMMs), lymphoma, mouse models and preclinical studies, preclinical (*in vivo*) studies

## Abstract

Diffuse large B cell lymphoma (DLBCL) is a highly aggressive form of lymphoma that is genetically and phenotypically diverse. Most DLBCLs arise from the germinal center and transcriptionally represent either germinal center or post-germinal center activated B cells by gene expression profiling. Recently, a revised classification of DLBCLs has identified at least six molecularly defined groups based on their genomic landscape. In this review, we discuss the use of transgenic animal models to study DLBCLs based on the more recent genetic-molecular classification framework. We highlight the importance and insights gained from hallmark animal models of DLBCL, containing over 60 different alleles, and their use in therapeutic discovery.

## Introduction

Diffuse large B cell lymphoma (DLBCL) is the most prevalent human lymphoid malignancy and accounts for up to 35% of all non-Hodgkin lymphomas (1). Two subtypes of DLBCLs were previously identified based on their gene expression resemblance to either germinal center B cells (GCB-DBLCL) or post-germinal center activated B cells (ABC-DLBCL) (1). GCB-DLBCL and ABC-DLBCL also differ in their response to chemotherapy and targeted agents. In order to understand why these tumors behave differently and to find shared therapeutic vulnerabilities for improving treatment responses, DLBCLs were further classified into six molecularly defined subtypes based on shared genomic abnormalities: EZB (*EZH2* mutations and *BCL2* translocation), ST2 (*SGK1* and *TET2* mutations), BN2 (*BCL6* fusion, *NOTCH2* mutations), A53 (*TP53* mutations), N1 (*NOTCH1* mutations) and MCD (*MYD88* and *CD79B* mutations) (2). MCD and N1 share features reminiscent of ABC-DLBCL, whereas the EZB and ST2 groups are closely related to GCB-DLBCL (3). The differences in gene mutations found in each subtype may translate into substantial differences in patient survival and therapeutic possibilities (3).

Traditional classification of DLBCL stems from our understanding of normal B cell development and differentiation. B cell development begins in the bone marrow, where a committed B cell undergoes immunoglobulin assembly by V, D, J recombination and a step-wise expression of the immunoglobulin heavy (IgH) and light (IgL) chains that form the B cell receptor (BCR) (4). Once a B cell completes BCR expression, it migrates into the periphery and takes up residency in secondary lymphoid organs such as the spleen or lymph nodes. Upon antigen activation, mature B cells enter germinal centers (GCs), highly dynamic microstructures that are formed transiently, with the aim of producing high-affinity antibodies and differentiating into antibody-secreting cells (5). Within the GC, B cells recirculate between the dark zone (DZ) and the light zone (LZ), two anatomically distinct microenvironments. Dark zone B cells (centroblasts) are highly proliferative and exhibit a high rate of somatic hypermutation to modify immunoglobulin variable regions. These cells then cease proliferation and transit into light zone B cells (centrocytes), where they are selected by antigen retained on follicular dendritic cells (FDCs), and compete for help from T-follicular helper cells (Tfh) (5). B cells with high affinity towards the antigen successfully receive T cell help through CD40L-CD40 interaction, and undergo class-switch recombination (CSR) through AID-dependent DNA double-stranded break to confer specific antibody function (5).

Most DLBCLs originate from germinal centers (GCs) as a result of the accumulation of multiple mutations. Normally, key checkpoints are in place to safeguard the normal process of a germinal center. These can be organized into three broad categories: 1) checkpoint markers that regulate GC entry, exit, and migration, 2) regulators of cell death and proliferation, and their associated signaling pathways, and 3) enzymes and transcription factors that govern structural genomic modification that turn-on or turn-off key regulatory gene expression. Detailed molecular mechanisms of these checkpoints are reviewed elsewhere (5). Mutations can hijack these key checkpoints and cause transformation of normal GC cells into malignant diseases such as Burkitt lymphomas (BL), Follicular lymphomas (FL), Marginal zone lymphoma (MZL), and DLBCLs. While the mechanisms behind why GC B cells acquire these mutations are diverse and incompletely understood, erroneous chromosomal translocation and aberrantly targeted somatic hypermutation remain two key events in the process. This is exemplified by AID-mediated CSR involving *MYC* translocation in Burkitt lymphoma (6, 7) and aberrant somatic hypermutation (SHM) that target gene loci, such as the *BCL6* promoter, resulting in its constitutive expression in GCB-DLBCL (8, 9).

Understanding how individual mutations disrupt the regulation of a normal B cell is key to studying its transformation mechanisms and to therapeutic discovery. In the clinics, this is limited by not enough patients to represent every combination of gene mutation. Therefore, establishing pre-clinical models that accurately represent the genetic events of DLBCLs *in vivo* is a significant objective in translational lymphoma research. Here, we review hallmark genetically engineered mouse models classified based on shared alterations in molecular pathways.

## B-cell-specific Cre-LoxP mouse models

**Figure 1:**
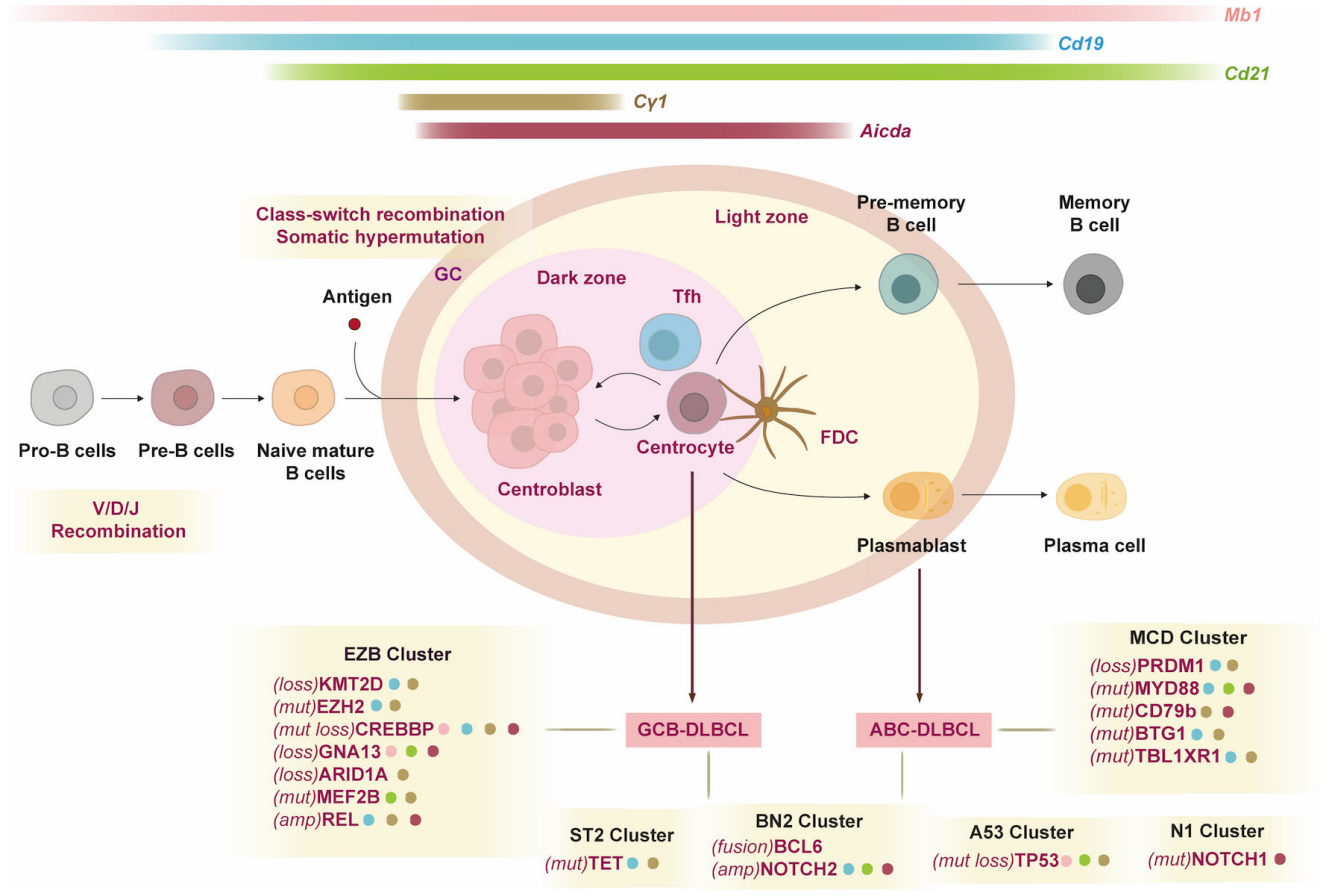
B cell specific Cre expression and DLBCL gene aberrations. Schematic of the major GC B cell developmental trajectory from an antigen-activated naïve mature B cells into either memory B cells or plasma cells. Genes highlighted above (i.e., *Mb1, Cd19, Cd21, Cγ1*, and *Aicda*), each marked by a different color indicative of their activity throughout different B cell differentiation stage, frequently used to drive Cre expression in modelling lymphoma development in mice. Highlighted boxes indicate the molecular subtype of lymphoma that originates from GC B cells, including the gene aberrations involved and the Cre-dependent mouse models employed for their aberration (i.e., loss of *KMT2D* in EZB cluster GCB-DLBCL modelled by *Cd19^Cre^* and *Cγ1^Cre^*). GC, germinal center; Tfh, T follicular helper; FDC, follicular dendritic cell.

The Cre-LoxP system enables targeted Cre recombinase–mediated DNA recombination at LoxP sites (10, 11). Cre recombinase, derived from bacteriophage P1, recognizes a 34-base-pair LoxP sequence and mediates site-specific excision, inversion, or translocation of the intervening DNA sequence depending on the LoxP orientation (11). In mice, multiple B cell conditional Cre models have been developed by fusing the *Cre* gene to promoters of B cell specific genes to drive their expression during different B cell developmental stages (**Figure 1**).

One of the earliest expressed genes in committed B cell lineages and used in gene targeting in mouse B cells is the *Mb1* gene. *Mb1* encodes the Ig-α (CD79A) subunit of the B cell receptor and is ubiquitously expressed from as early as the pro-B cell stage (12). The *Mb1^Cre^* mouse strain was developed by replacing exon 2 and exon 3 of the *Mb1* gene with a human Cre cDNA (13). Mice carrying *Mb1^Cre^* display over 90% recombination efficiency in early pre-B cells. This allows the *Mb1^Cre^* mice to be effectively used for studying genetic events associated with early B cell development (13). Another widely used mouse model with B cell specific Cre protein expression is the *Cd19^Cre^* mice. CD19 is a transmembrane glycoprotein expressed in pre-B cells through to non-terminally differentiated mature B cells. *Cd19^Cre^* lines were generated by gene-targeting of *Cd19* exon 2 with hemizygous Cre expression (14). Although both *Mb1* and *Cd19* show early B cell expression, *Mb1^Cre^* mice show enhanced early B cell recombination activity in the bone marrow compared to *Cd19^Cre^* mice, which show low efficiency in the bone marrow but increased efficiency in peripheral mature B cells. Complementary to the mature B cell expression of *Cd19^Cre^*, a *Cd21^Cre^* strain was engineered by transgenic expression of Cre under the *Cd21* promoter. *Cd21^Cre^* mice display efficacy from transitional to naïve mature B cells (15). An additional advantage of *Cd21^Cre^* owns to the lack of competitive disadvantage compared to *Cd19^Cre^* mice expressing only one allele of *Cd19* (14), making the *Cd21^Cre^* mice particularly attractive for studying gene function in mature B cells.

Germinal center (GC) B cells upregulate AID for SHM and CSR (16). Consequently, AID-driven Cre (*Aicda^Cre^*) and Cγ1-driven Cre (*Cγ1^Cre^*) have been developed to enable targeted gene modification specifically in GCB cells. *Aicda^Cre^* mice were generated by replacing exon 1 of the *Aicda* gene with Cre-expressing cassettes and *Cγ1^Cre^* mice were developed in by utilizing *Cre* cDNA fused into the *Cγ1* locus, thereby enabling the tracking of B cell antibody class-switching to IgG upon immunization (17, 18). Worth noting is that both *Cγ1^Cre^* and *Aicda^Cre^* function are likely to require immunization with a T-dependent antigen that triggers GC response and formation. This is particularly important when a continuous supply of antigen is required for boosting germinal center reaction in studying tumor suppressor function in GC B cells.

These B cell specific *Cre* expressing animals have been widely used in studying the pathogenesis of lymphomas of various cell of origin. In this review, we highlight hallmark *Cre-*driven lymphoma models grouped on the basis of common genetic features (**Table 1**; **Figure 1**).

**Table 1:** Overview of B cell-specific aberrations in modeling DLBCLs.

Cluster	Gene	Genetic model	Immunization	Lymphoma penetrance	Reference
EZB	*KMT2D*	*Kmt2d^fl/fl^Cd19^Cre^*	SRBC	N/A	(19)
		*Kmt2d^fl/fl^Cγ1^Cre^*		0/15	
		*Kmt2d^fl/fl^ Cγ1^Cre^VavP-Bcl2*		22/28	
	*EZH2*	*Ezh2^Y641fl/+^Cd19^Cre^*	Not immunized	15/15	(20)
		*Ezh2^fl/fl^ Cγ1^Cre^*	SRBC NP-KLH	N/A	(21)
		*Ezh2^Y641N^ Cγ1^Cre^*		N/A	
		*Ezh2^Y641N^ Cγ1^Cre^VavP-Bcl2*		7/10	
		*Ezh2^fl/fl^ Cγ1^Cre^*	SRBC	N/A	(22)
		*Ezh2^Y641F/+^ Cγ1^Cre^*		0/4	
		*Ezh2^Y641F/+^ Cγ1^Cre^ Iμ-Bcl6*		10/12	
	*CREBBP*	*Crebbp^fl/+^ Mb1^Cre^*	Not immunized	4/37	(23)
		*Crebbp^fl/fl^ Mb1^Cre^*		5/27	
		*Crebbp^fl/fl^Mb1^Cre^Eμ-Bcl2*		5/29	
		*Crebbp^fl/fl^Cd19^Cre^*	Not immunized	2/17	(24)
		*Crebbp^fl/fl^Cγ1^Cre^*	SRBC NP-KLH	3/22	
		*Crebbp^fl/fl^ Cγ1^Cre^VavP-Bcl2*		22/24	
		*Crebbp^fl/+^Aicda^Cre^*	SRBC	0/13	(25)
		*Crebbp^fl/fl^Aicda^Cre^Eμ-Myc*		Lymphoma observed, penetrance not quantified	
	*KMT2D & CREBBP*	*Crebbp^fl/+^Cγ1^Cre^* *Kmt2d^fl/+^Cγ1^Cre^*	SRBC NP-KLH	N/A	(26)
		*Kmt2d^fl/+^Crebbp^fl/+^Cγ1^Cre^*		N/A	
		*Crebbp^fl/+^ Cγ1^Cre^ VavP-BCL2* *Kmt2d^fl/+^ Cγ1^cre/+^ VavP-BCL2* *Crebbp^fl/+^Kmt2d^fl/+^ Cγ1^Cre^ VavP-BCL2*	SRBC	Lymphoma observed, penetrance not quantified	(27)
	*GNA13*	*Gna13^fl/fl^ Mb1^Cre^*	Not immunized	10/18	(28)
		*Gna13^fl/fl^ Aicda^Cre^*	SRBC	N/A	(29)
		*Gna13^fl/fl^Rosa26Stop^FL-Myc^Aicda^Cre^*		4/4	
		*Gna13^fl/+^Cd21^Cre^*	SRBC	8/23	(30)
		*Gna13^fl/fl^Cd21^Cre^*		27/34	
		*Gna13^fl/fl^Aicda^Cre^*		N/A	
		*Gna13^fl/fl^Aicda^Cre^ Rosa26^LSL-Cas9/+^*		N/A	
	*ARID1A*	*Arid1a^fl/+^Cγ1^Cre^* *Arid1a^fl/+^Cγ1^Cre^VavP-Bcl2*	SRBC NP-KLH NP-OVA	Lymphoma observed, penetrance not quantified	(31)
	*MEF2B*	*Mef2b^+/stopD83V^Cd21^Cre^*	Not immunized	14/47	(32)
		*Mef2b^+/stopD83V^Cd21^Cre^BCL2^tg^*		19/21	
		*Mef2b^fl/+^Cγ1^Cre^* *Mef2b^fl/fl^Cγ1^Cre^* *Mef2b^fl/stopD83V^Cγ1^Cre^*	SRBC	N/A	
		*Mef2b^P297L/+^Cγ1^Cre^*	SRBC	5/34	(33)
		*Mef2b^P297L/P297L^Cγ1^Cre^*		4/21	
		*Mef2b^P297L/P297L^Cγ1^Cre^ Bcl2-Ig^Tg^*		13/14	
	*REL*	*Rel^fl/fl^Cd19^Cre^*	Not immunized	N/A	(34)
		*Rel^fl/+^Cγ1^Cre^* *Rel^fl/fl^Cγ1^Cre^*	SRBC NP-KLH	N/A	
		*Rel^OE^ Aicda^Cre^*	SRBC	N/A	(35)
ST2	*TET2*	*Tet2^fl/fl^Cd19^Cre^*	SRBC	N/A	(36)
		*Tet2^fl/fl^Cγ1^Cre^*		0/7	
		*Tet2^fl/fl^Cγ1^Cre^;IμBcl6*		12/12	
BN2	*BCL6*	*IμBcl6*	SRBC	27/79	(37)
	*NOTCH2*	*Notch2^fl/fl^Cd21^Cre^* *Notch2^fl/fl^Aicda^Cre^*	NP-OVA NP-CGG	N/A	(38)
		*Notch2^Trunc^Cd19^Cre^*	NP-KLH	Lymphoma observed, penetrance not quantified	(39)
		*Notch2^Trunc^Spen^Lof^Cd19^Cre^*			
A53	*TP53*	*Trp53^fl/fl^Mb1^Cre^*	Not immunized	100% penetrance, not quantified	(40)
		*Trp53^fl/+^Cd21^Cre^* *Trp53^fl/fl^Cd21^Cre^*	SRBC	10/20	(41)
		*Trp53^fl/fl^Cγ1^Cre^*	Not immunized	N/A	(42)
		*Xrcc4^fl/fl^Trp53^fl/fl^Cγ1^Cre^*		11/47	
N1	*NOTCH1*	*Nicd1^Tg^Aicda^Cre^*	Not immunized	N/A	(43)
MCD	*MYD88*	*Myd88^p.L252P^Cd19^Cre^*	Not immunized	1/4	(44)
		*Myd88^p.L252P^Cd21^Cre^*		1/3	
		*Myd88^p.L252P^Aicda^Cre^*	SRBC	1/3	
		*Myd88^p.L252P^Rosa26^LSL.BCL2.IRES.GFP^Cd19^Cre^*	Not immunized	7/8	
		*Myd88^p.L252P^Cd79b^Y196H^ Cd21^Cre^*	SRBC	N/A	(45)
		*Myd88^p.L252P^Cd79b^Y196H^Prdm1^fl/+^Cd21^Cre^*			
		*Myd88^p.L252P^Cd79b^Y196H^Prdm1^fl/+^* *Bcl2^OE^Aicda^Cre^*	Not immunized	5/8	
	*PRDM1*	*Prdm1^fl/fl^Cd19^Cre^*	Not immunized	4/38	(46b)
		*Prdm1^fl/fl^C*γ*1^Cre^*	SRBC	3/25	
		*Prdm1^fl/fl^Ikk2ca^stopF/stopF^C*γ*1^Cre^*	SRBC	5/5	(47)
		*Prdm1^fl/fl^Ikk2caGFP^stopF/stopF^Cγ1^Cre^* *Prdm1^fl/fl^p53^fl/fl^IIkk2caGFP^stopF/stopF^Cγ1^Cre^*	SRBC	Lymphoma observed, penetrance not quantified	(48)
	*BTG1*	*R26^lsl.Btg1Q36H/+^Cd19^Cre^*	SRBC NP-KLH NP-OVA NP-Ficoll	N/A	(49)
		*R26^lsl.Btg1Q36H/+^Cγ1^Cre^*		N/A	
		*R26^lsl.Btg1Q36H/+^Cγ1^Cre^VavP-Bcl2*		Lymphoma observed, penetrance not quantified	
	*TBL1XR1*	*Tbl1xr1^fl/fl^Cγ1^Cre^*	SRBC	N/A	(50)
		*Tbl1xr1^D370Y^Cγ1^Cre^*		N/A	
		*Tbl1xr1^fl/fl^Cd19^Cre^VavP-Bcl2*		5/5	
		*Tbl1xr1^fl/fl^Cγ1^Cre^VavP-Bcl2*		N/A	

SRBC, sheep red blood cell; NP-KLH, 4-Hydroxy-3-nitrophenylacetyl conjugated to Keyhole Limpet Hemocyanin; NP-OVA, 4-Hydroxy-3-nitrophenylacetyl conjugated to ovalbumin; NP-Ficoll, 4-Hydroxy-3-nitrophenylacetyl conjugated to ovalbumin conjugated to Ficoll; N/A, not applicable for tumor penetrance study – models used for studying gene function but mice are not analyzed for tumor development.

## EZB subtype

EZB-cluster DLBCLs are characterized by *EZH2* mutation, *BCL2* translocation, *REL* amplification, and closely resemble features of GCB-DLBCL (3). The EZB cluster also shows frequent inactivation of the tumor suppressors *KMT2D*, *CREBBP*, and frequently show disruption of germinal center signaling, such as GNA13, JAK-STAT and PI3K pathways. In addition, EZB cluster can be further classified into *MYC^+^* or *MYC^-^* subtypes based on their resemblance to GC light-zone B cells (EZB-MYC^-^) or dark-zone B cells (EZB-MYC^+^). These shared features make EZB-tumors likely targets for EZH2, BCL2, and PI3K inhibitors.

### EZH2

Enhancer of zeste homolog 2 (EZH2) functions as part of the polycomb repressive complex (PRC2) and catalyzes H3K27 methylation at chromatin enhancer regions (51). Approximately 30% of GCB-DLBCLs have *EZH2* mutation, and most result in a gain-of-function Y646F (mouse Y641F) substitution in its catalytically active SET domain (52). EZH2 is highly expressed in germinal center B cells to regulate genes involved in controlling proliferation checkpoints and in plasma cell differentiation (21, 53). *Ezh2^Y641F^Cγ1^Cre^* mice display heightened GC response following sheep red blood cell (SRBC) immunization. *Ezh2^Y641F^Cγ1^Cre^* B cells are enriched for H3K27me3 at gene targets that play a role in enhancing differentiation block while repressing cell cycle checkpoints. Lymphomagenesis by *Ezh2^Y641F^* is *Bcl6* and *Bcor* dependent (22). In an adoptive transfer experiment, recipients that receive donor B cells from *Ezh2^Y641F^Cγ1^Cre^IμBcl6* mice develop fully penetrating lymphoma or lymphoid hyperplasia upon continuous immunization over time (22). However, lymphomas do not develop in recipient animals transferred with *Ezh2^Y641F^Cγ1^Cre^* B cells lacking constitutive *Bcl6* expression. Mechanistically, mutated EZH2 cooperates with BCL6 to recruit a non-canonical PRC1/BCOR complex to repress genes associated with the proliferation checkpoint (e.g., *Cdkn1a*, *Cdkn1b*) and genes controlling germinal center exit (e.g., *Prdm1*, *Irf4*), thus allowing these cells to be locked in a relentless cell cycle in the GC (22). Targeting EZH2 with GSK343 cooperates strongly with anti-BCL6 drug FX1 in suppressing lymphoma growth (22), suggesting a potential action for EZH2 and BCL6 inhibitors as a combination therapy for these lymphomas.

### KMT2D

(MLL2) is a histone methyltransferase that catalyzes the transcriptional activating H3K4 methylation at gene enhancer regions (54). *KMT2D* is the most frequently mutated epigenetic modifier in DLBCL and is found in 30% of DLBCL patients (55). Pre-GC and GC-B cell-specific KMT2D-deficient mice have been generated by crossing a *Kmt2d* floxed allele with either *Cd19^Cre^* or *Cγ1^Cre^* mice (19). Early loss of KMT2D in *Kmt2d^fl/fl^Cd19^Cre^* mice boosts a three-fold expansion of GC B cells, indicating that loss of KMT2D enhances GC formation. B cells from these mice display loss of H3K4 methylation markers and enhanced cell cycle gene program and anti-apoptotic genes (19). On the other hand, deletion of KMT2D in GCB cells in *Kmt2d^fl/fl^Cγ1^Cre^* mice does not confer advantages in GC expansion upon immunization, indicating that KMT2D deletion conferred B cell advantage predominantly affects B cells prior to their entry into the germinal center by remodeling the epigenetic landscape of precursor cells that favors their GC entry. Indeed, compared to 221 differentially expressed genes in *Kmt2d^fl/fl^Cd19^Cre^* B cells, the number of differentially expressed genes in *Kmt2d^fl/fl^Cγ1^Cre^* B cells reduces to 60 (19). A core list of altered genes regulating cell cycle and resistance to apoptosis overlaps in *Kmt2d^fl/fl^Cd19^Cre^* and in *Kmt2d^fl/fl^Cγ1^Cre^* B cells, suggesting that earlier deletion of KMT2D may create a larger pool of cells that could enter the GC and serve as targets for additional lesions (19). Indeed, KMT2D loss alone is insufficient to cause lymphoma in both models. Instead, KMT2D deletion cooperates with BCL2 deregulation to drive FL and DLBCLs (19, 27).

### CREBBP

Another frequently mutated epigenetic modifier in DLBCLs is the histone acetyltransferase *CREBBP* and its homolog *EP300*. As the second most frequently mutated gene in DLBCL, inactivating mutations in the *CREBBP* are found in ~30% of lymphomas and are mostly enriched in the EZB cluster (52, 56). CREBBP controls a transcriptional network within the germinal center that is related to genes regulating cycle entry and terminal differentiation that are normally repressed by BCL6 (57) Loss of CREBBP in *Crebbp^fl/fl^Cd19^Cre^* mice and in *Crebbp^fl/fl^Cγ1^Cre^* mice results in significantly decreased plasma cell differentiation following sheep red blood cell immunization. Conversely, CREBBP loss causes a dose-dependent gain in proliferation advantage when these B cells are stimulated with anti-CD40 and IL-4 (24). CREBBP deficiency alone has very low penetrance in lymphomagenesis. However, crossing *Crebbp^fl/fl^Cγ1^Cre^* with *VavP-Bcl2* allele significantly increases lymphoma penetrance to over 90%, most resembling follicular lymphoma, which closely models the co-occurrence of these two gene lesions in over half of human follicular lymphomas (24). Aside from transcriptional regulation of cell proliferation and differentiation, deletion of CREBBP in the germinal center compartment of *Crebbp^fl/fl^Aicda^Cre^* mice results in a strong loss of MHC II expression in these cells (25). Similarly, although CREBBP deficiency alone does not cause lymphoma, but when crossed to the Eμ-MYC driven lymphoma mice, *Crebbp^fl/fl^Aicda^Cre^* significantly accelerates the formation of MYC-driven B cell lymphomas through increasing immune evasion by silencing MHC II (25, 58).

Loss-of-function mutations of *CREBBP* and *KMT2D* co-occur in ~30% of GCB-DLBCLs and up to 60% of follicular lymphoma (52), suggesting the co-selection of *CREBBP* and *KMT2D* mutations. Co-inactivation of the epigenetic modifiers CREBBP and KMT2D in *Crebbp^fl/+^Kmt2d^fl/+^Cγ1^Cre^* mice drives additional abnormal GC polarization, with additive effects in both GC cell number, GC size, and DZ to LZ ratio (26). Functionally, CREBBP and KMT2D co-occupy sets of genes that regulate BCR signaling, G-protein signaling, and transcription factors important for the GC program (e.g., *Bcl6*). This remarkable observation has been further recapitulated in a recent study, indicating that combined haploinsufficiency of CREBBP and KMT2D leads to GC fate decision error and confers the inactivation of immune synapse crosstalk in lymphoma development (27).

### GNA13

One of the most frequently deregulated cell surface receptors implicated in EZB-DLBCL lymphomagenesis is the GNA13 mutation (28). GNA13 mutation, heterozygous loss or homozygous deletion are found in 22.5% of EZB subtype lymphoma patients (3). *GNA13* encoded protein Gα13, together with its responding element sphingosine-1-phosphate receptor-2 (S1PR2), plays a non-redundant role in maintaining GC B cell niche and homeostasis by confinement of B cells in the GC follicle (59). Targeted disruption and deficiency of SIPR2 result in hyperphosphorylated AKT and aberrant B cell migration, contributing to imbalanced GC and DLBCL formation (59). *Gna13^fl/fl^Mb1^Cre^* animals develop aggressive lymphoma as a result of germinal center B cells outgrowth and loss of their GC conferment together with aberrant AKT activation (28). Additionally, *Gna13*-deficient B cells are resistant to apoptosis and are less dependent on T cell help to sustain GC expansion. *GNA13* mutations and *BCL2* translocations frequently occur together in GCB-DLBCLs. Combining GNA13 deficiency with BCL2 protein overexpression in *Gna13^fl/fl^Mb1^Cre^EμBcl2* mice significantly increases GC B cell outgrowth and dissemination (28). Aside from its role in restricting GC-derived B cell lymphomas, *Gna13* deficiency in mature B cells specifically augments lymphoma growth in mesenteric lymph nodes (mLNs) (30). *Gna13^fl/fl^Cd21^Cre^* mice develop lymphoma in the mLNs between 10 and 25 months due to aberrant activation of mTORC1 and MYC. These findings imply that targeting AKT-mTORC signaling pathways may have therapeutic benefits for disseminated DLBCLs with GNA13 loss-of-function mutations.

### ARID1A

A catalytic subunit of the canonical BAF complex, is frequently altered by loss-of-function mutations across multiple cancer types (60, 61). In DLBCL, *ARID1A* truncation mutations are enriched in the EZB cluster, comprising ~23% of cases (3). *Arid1a^fl/+^Cγ1^Cre^* mice exhibit dose-dependent impairment in germinal center B cell expansion upon SRBC immunization. This is accompanied by an increased proportion of centroblasts vs centrocytes within the GC, suggesting likely early GC exit and differentiation (31). Indeed, the absence of ARID1A shifts GC cell fate to a memory B cell phenotype, which more readily re-enters the GC upon secondary antigen encounter, causing heightened secondary GC response (31). Long-term follow-up of mice reconstituted with *Arid1a^fl/+^Cγ1^Cre^* bone marrow cells followed by SRBC immunization shows the absence of lymphoma onset despite GC enlargement. However, ARID1A deficiency strongly cooperates with BCL2 translocation in *Arid1a^fl/+^Cγ1^Cre^VavP-Bcl2* mice to drive follicular lymphoma and transformation to DLBCL (31). cBAF inhibition induces strong apoptosis in lymphoma cells with a single allele ARID1A missense mutation, suggesting a possible therapeutic opportunity for ARID1 mutant lymphomas by targeting cBAF.

### MEF2B

(myocyte enhancer-binding factor 2B), a transcriptional regulator that directly transactivates BCL6, is associated with GCB-DLBCL pathogenesis through deregulating BCL6 function (62). *MEF2B* is mutated in ~15% of follicular lymphoma and diffuse large B cell lymphomas. *MEF2B^D83V^* somatic mutation is enriched in EZB lymphomas (3). *Mef2b^fl/fl^Cγ1^Cre^* mice show dose-dependent reduction in GC formation and in DZ cell number. *Mef2b^fl/fl^Cγ1^Cre^* germinal center B cells display a consistent reduction in BCL6 expression, consistent with BCL6’s role in instructing GC B cell differentiation (62). Contrary to the loss of MEF2B, *Mef2b^D83V^Cγ1^Cre^* mice display GC enlargement and an accumulation of LZ cells (32). This is partly due to escaping negative regulation by components of the HUCA complex and HDACs and thereby increasing transcriptional activity in genes including BCL6 targets and also genes involved in BCR and NFκB signaling (32). However, the MEF2B mutation has relatively weak oncogenic activity with <30% of *Mef2b^D83V^Cd21^Cre^* mice developing tumors, despite turning on *Mef2b^D83V^* mutation earlier in mature B cells. Notably, *MEF2B* mutation frequently co-occurs with *BCL2* rearrangements to drive lymphomagenesis (63). Indeed, crossing *Mef2b^D83V^Cd21^Cre^* with *Bcl2 Tg* mice results in more aggressive features of tumors with nearly complete penetrance, accompanied by early onset and heightened tumor dissemination, suggesting a strong cooperation between these two gene lesions (32).

### REL

(cRel), a subunit of the Rel/NFκB transcription factor family, is exclusively targeted by amplification in 34% EZB-DLBCLs (3). *Rel^fl/fl^Cd19^Cre^* B cells show significant impairment in GC formation following SRBC immunization due to failure to increase cell metabolism that directs germinal center B cell proliferation (34). On the other hand, *Aicda^Cre^*-mediated *Rel* overexpression mice display long-term GC persistence following immunization and enhanced GC re-entry following secondary challenge, accompanied by increases in immunoglobulin class switching (35). This observed increase in GC re-entry provides a precursor pool of pre-malignant B cells that accumulate secondary mutations in the germinal center such as EZH2, KMT2D or CREBBP that cooperate with other translocation events that lead to lymphoma development. This is consistent with a more recent study demonstrating that REL amplification is associated with blockade in B cell differentiation into memory cells, highlighting an additional function of this amplicon in blocking terminal differentiation of B cells during lymphomagenesis (64). Given that *REL* amplification is associated with metabolic fitness of cells within the GC, it might be plausible to target the AKT-MYC axis for lymphomas with increased REL expression.

## ST2 subtype

ST2 groups is defined by recurrent mutations in SGK1 and TET2 and mostly represent a subclass of GCB-DLBCL with dysregulations of B cells from the GC light zone (3). Its malignant process often involves glycolytic pathway dysregulation with the PI3K/AKT and JAK2 kinase pathways affected. While SGK1 mutations represent 37% of ST2 subtype, a genetic model for this mutation is missing. Therefore, this section below will focus on TET2 mutation.

### TET2

(ten-eleven translocation) enzymes oxidize 5-methylcytosine, a key step in DNA demethylation (65). Approximately 48% of ST2 cluster DLBCLs have *TET2* mutations (3). Deletion of TET2 in B cells using *Tet2^fl/fl^Cd19^Cre^* mice induces GC hyperplasia following SRBC immunization (36). Notably, TET2 deletion causes an increase in GC size rather than the number of GCs per spleen. This is due to impaired plasma cell differentiation following TET2 deletion, indicating that TET2 is required for proper GC exit rather than its initiation (36). *Tet2^fl/fl^Cd19^Cre^* mice have particular accumulation towards centrocytes and lower expression of transcription factors such as PRDM1 and IRF4, consistent with the observed failure in GC exit (36). Crossing *Tet2^fl/fl^Cγ1^Cre^* to *IμBcl6* mice with constitutive BCL6 expression accelerates lymphoma development with a striking 100% penetrance (36). However, *Tet2^fl/fl^Cγ1^Cre^* mice alone do not develop lymphoma, suggesting that TET loss acts as a tumor suppressor in BCL6-driven lymphoma. Gene signatures in TET*-*deficient GCs remarkably overlap those observed by CREBBP inactivation (36), suggesting an overlapping mechanism of action. CREBBP target enhancers require TET2-mediated DNA 5hmC. Therefore, it would be predicted that lymphomas with TET2 deficiency may be targeted with HDAC inhibitors.

## BN2 subtype

BN2 is a unique cluster of DLBCL that possesses features of ABC-DLBCL, GCB-DLBCL, and unclassified DLBCL (3). 72% of BN2 tumors have *BCL6* translocation. BCL6 interacts with EZH2, CREBBP, MEF2B, and among other genes associated with the EZB cluster described above. This phenotype underpins that the genetic themes from BN2 and EZB may potentially cooperate in driving GCB-DLBCL. Aberrantly activated NOTCH2 signaling that cooperates with BCR-dependent NFκB survival signaling, distinguishing BN2 from EZB and provides a unique therapeutic opportunity.

### BCL6

Is required for GC formation and sustaining B cell proliferation within the GC by repressing transcriptional targets that instruct cell cycle arrest and terminal differentiation (e.g., *PRDM1*) (66). Constitutive *BCL6* expression in DLBCL is driven mainly as a result of erroneous somatic hypermutation targeting its 5’ regulatory region or class-switch recombination fusing the *BCL6* gene to the immunoglobulin gene (8). To model translocation events, a full-length mouse BCL6 coding sequence is fused downstream of the immunoglobulin heavy chain (IgH) Iμ promoter (*IμBcl6*). Young *IμBcl6* mice display an increased number of GC at both steady state and post-immunization. This is accompanied by a reduction in the number of plasmablasts and plasma cells (37). When aged to 6 months, *IμBcl6* mice develop lymphoproliferative disease that progressively evolves towards clonal lymphoma by 13 months. BCL6 translocation and/or hypermutation sustains its expression in the germinal center and cooperates with CREBBP and its homolog P300 for lymphomagenesis, which is described above and reviewed extensively elsewhere (56, 67). Inhibiting BCL6 by inhibitors (e.g., FX1) are effective in killing lymphomas that are dependent on BCL6 for survival *in vitro* (37) and also drastically reduces GC expansion in immunized animals (68).

### NOTCH2

(NOTCH Receptor 2) encodes a conserved protein that belongs to the Type 1 transmembrane Notch family. *NOTCH2* truncation and amplification are found in approximately 40% of BN2-cluster DLBCL cases (3). NOTCH2 signaling governs B cell homing to the marginal zone and activated B cell differentiation towards memory B cells, but not GC B cells and plasma cells (38). Inactivation of NOTCH2 signaling in *Notch2^fl/fl^Cd21^Cre^* mice impairs BCR signaling required for memory B cell survival while *Notch2^fl/fl^Cd19^Cre^* mice lack marginal zone B cells (38). *Notch2^Trunc^Cγ1^Cre^* with human *NOTCH2* truncated intracellular domain knock-in displays a reduction in splenic B cell population compared to matched controls and impaired germinal center formation following NP-CGG immunization (39). To monitor extrafollicular development of B cells with NOTCH2 truncation, *Notch2^Trunc^Cd19^Cre^* displays enhanced marginal zone B cell skewing. Both *Notch2^Trunc^Cγ1^Cre^* and *Notch2^Trunc^Cd19^Cre^* B cells are blocked from differentiation into plasmablast and plasma cells, which potentiates these B cells from malignant transformation (39).

BN2 tumors also frequently harbor truncating mutations in the transcription repressor SPEN (52). Combining *Notch2^Trunc^* with *Spen^Lof^* in *Cd19^Cre^* driven mice develops lymphomas that highly resemble marginal zone lymphoma and DLBCLs, consistent with a preference towards marginal zone cell development instructed by NOTCH2 signaling and that BN2 lymphomas represent transformation of MZLs (39). Interestingly, lymphomas develop more frequently in female animals and show greater response to TLR7 agonists as well as increased TLR7 expression, suggesting that *Notch2^Trunc^* augments aberrant TLR7 signaling. Thus, targeting IRAK1/4 downstream of TLR7 may be considered as a novel approach in BN2 lymphomas harboring NOTCH2 mutation.

## A53 subtype

A53 is characterized by TP53 inactivation or DNA damage leading to cell aneuploidy. Over 90% of A53 tumors have mutations in *TP53* or chromosomal abnormalities associated with *TP53* inactivation (3). The majority of A53 abnormalities are associated with ABC-DLBCL. Aside from *TP53* inactivation, *B2M* inactivation by truncation of homozygous deletion is present in ~35% of A53 tumors, suggesting a role for immune evasion. However, the role of lymphoma immune microenvironment is emerging and discussed later.

### TP53

(*Trp53* in mouse) encodes the transcription factor p53 that functions as a tumor suppressor gene by controlling genes regulating cell cycle and apoptosis (69). *TP53* mutation, heterozygous loss, and homozygous deletion are found in nearly 90% of the A53 cluster DLBCL cases (3). *Trp53* null mice develop lethal lymphoma and leukemia that cooperate with both BCL2 or MYC overexpression (70). These earlier studies with germline loss of p53 often develop different types of malignancies in other tissues. Inactivation of p53 at an earlier developmental stage in *Trp53^fl/fl^Mb1^Cre^* results in lethal lymphoproliferative disease that involves cells from multiple B cell lineages, including transformation of both pro-B cells and mature B cells (40). B cell tumor onset in *Trp53^fl/fl^Mb1^Cre^* mice is similar to that observed in *Trp53-/-* animals. Presumably that *Trp53^fl/fl^Mb1^Cre^* B cells equally acquire other lesions and oncogenic translocations initiated by RAG/AID-mediated gene translocation (41). Conditional inactivation of p53 in *Trp53^fl/fl^Cd21^Cre^* animals provides another model to study the consequence of p53 deletion in mature B cells (41). These mice develop lethal lymphoma between 8 and 12 months of age (41). *Trp53^fl/fl^Cd21^Cre^* B cell lymphomas are mostly IgM-positive with limited somatic hypermutation, suggesting a cell of pre-germinal center origin and resembling splenic marginal zone lymphomas (41). *Trp53^fl/fl^Cd21^Cre^* lymphomas also frequently carry translocation events involving the *IgH* locus as a result of mis-repair of non-homologous end joining (NHEJ) or double-stranded breaks (DSB). However, *Myc* translocation is rare in these tumors, suggesting other mechanisms as a result of *Trp* deficiency caused by genome instability. Indeed, deletion of the non-homologous end-joining protein XRCC4 with p*53* in *Xrcc4^fl/fl^Trp53^fl/fl^Cγ1^Cre^* animals results in the development of B cell lymphoma that are GC origin with high levels of clonal heterogeneity for *Myc* translocation (42).

## N1 subtype

N1 represents a small subset of quiescent ABC-DLBCL dominated by a gain-of-function *NOTCH1* mutation, similar to those in chronic lymphocytic leukemia (CLL) and mantle cell lymphoma (MCL) (3). Additionally, these tumors also acquire mutations that dysregulate NFκB pathway to drive B cell differentiation. Tumor microenvironment of N1 exhibited expansion of immunosuppressive-related cell types, including regulatory T cells, resulting in the overall least favorable in patient survival probability (3).

### NOTCH1

Belongs to the NOTCH receptor family that is associated with diverse roles in lymphocyte development and differentiation, and NOTCH1 has a major effect on T cell development (71, 72). Genetic alterations in *NOTCH1* occur in a wide range of B cell malignancies, including mantle cell lymphoma, N1-cluster DLBCL, and FL (3, 73, 74). N1 DLBCLs display gene signatures that resemble memory B cells, suggesting post-GC origin. Additionally, N1 subtype expresses prominent signatures of multiple immune cell populations. Most N1 DLBCL mutations are truncation mutations localized in the PEST domain, causing aberrant activation of NOTCH signaling (52). Conditional expression of an active form of NOTCH1 in GC B cells using *Nicd1^Tg/-^Aicda^Cre^* displays normal B cell development but increased GC expansion (43). Expansion of GC is accompanied by a decrease in apoptosis in *Nicd1^Tg/-^Aicda^Cre^* mice, countering the high rate of cell death experienced by normal GC B cells, consistent with a previous report (75). Nevertheless, aged *Nicd1^Tg/-^Aicda^Cre^* mice do not develop B cell neoplasm. These mice display heightened regulatory T cell and type 2 T helper cell responses, attributing to extracellular IL-33 release by B cells (43). This immune privilege is consistent with the genomic data from N1 DLBCL, exhibiting a unique tumor microenvironment that enables immune evasion (43). Given that NOTCH1 mutation only occurs in N1 cluster DLBCL, NOTCH1 mutation therefore may contribute to N1 DLBCL in two ways: 1) promoting germinal center B cell survival, and 2) promoting immune evasion. Therefore, this model is particularly useful for studying immune checkpoint inhibitors or targeting of Treg cells for the treatment of lymphomas.

## MCD subtype

MCD subtype DLBCL cases almost exclusively represent ABC-DLBCL with a small fraction of unclassified DLBCL (52). Over 80% MCD tumors have *MYD88^L265P^* or *CD79B* aberration and 40% harbor both mutations. Signaling molecules linking the B cell receptor to NFκB are frequently aberrant in MCD tumors but not in other tumors. To block plasmacytic differentiation driven by chronic NFκB activation, MCD tumors frequently harbor inactivation mutation in *PRDM1*. MCD tumors also have significantly higher *BCL2* expression compared to other tumors, targeted by both genomic amplification and translocation. These subtypes of tumors are thus candidates for drugs acting downstream of the B cell receptor such as BTK, PI3K inhibitors and drugs targeting pro-survival signaling.

### MYD88

Is an adaptor protein downstream of Toll-like receptor signaling that activates the NFκB pathway. Exome analysis of 574 DLBCL patient biopsies identifies MYD88 mutations in 39% of ABC-DLBCL but not GCB-DLBCL patients (76). These mutations predominantly involve the hotspot p.L265P variant, which is therefore regarded as a key driver of the constitutive NFκB program in ABC-DLBCL (76). Mice with B cell specific *Myd88^p.L252P^Cd19^Cre^* develop lymphoproliferative disease from ~60 weeks old (44). This is also observed when *Myd88^p.L252P^* is expressed at a later B cell developmental stage in *Myd88^p.L252P^Cd21^Cre^* and in *Myd88^p.L252P^Aicda^Cre^* mice (44). Morphologically, these mice resemble clinical lymphoproliferative disease with only the occasional development of lymphoma (44). Given that ABC-DLBCLs have higher *BCL2* expression, a conditional BCL2 overexpression cassette is also introduced. *Myd88^p.L252P^Rosa26^LSL.BCL2.IRES.GFP^Cd19^Cre^* mice develop splenomegaly substantially earlier than *Myd88^p.L252P^Cd19^Cre^* mice (44). Additionally, these mice develop clonal or oligoclonal lymphomas that express post-GC markers such as Irf4 and Cd138 and are entirely negative for Bcl6 (44).

Patients with M*YD88^L265P^* frequently carry *CD79B* mutations. Retroviral overexpression of *Myd88^p.L252P^* together with *Cd79b^Y196H^* increases B cell expansion and differentiation compared to *Myd88^p.L252P^* alone (45). Consistently, *Myd88^p.L252P^ Cd79b^Y196H^ Cd21^Cre^* mice exhibit stronger outgrowth of germinal center B cells and stronger differentiation into plasmablasts and plasma cells. To better recapitulate the genomic features of MCD tumors, of which approximately one-third have loss of Prdm1, a *Myd88^p.L252P^Cd79b^Y196H^Prdm^1f/+^Cd21^Cre^* model is generated (45). Heterozygous loss of *Prdm1* in these mice causes a 10-fold competitive advantage in spontaneous GC expansion compared to control animals. These cells are strongly enriched in the GC dark zone and display enhanced proliferation as well as increased Irf4 expression (45). Crossing these animals to alleles that conditionally overexpress Bcl2 to the GCs in *Myd88^p.L252P^Cd79b^Y196H^Prdm^1f/-^Bcl2^OE^Aicda^Cre^* mice counters apoptosis of GCBs and further increases their expansion to over 50-fold (45). These mice have dramatically increased in both the size and number of GC compared with all other genotypes and develop DLBCL at a much younger age of 8 months. Collectively, these results demonstrate the cooperation of hallmark mutations surrounding oncogenic MYD88 signaling and the possibility for targeting these for therapeutic intervention.

### BLIMP1

A zinc-finger-containing transcriptional repressor encoded by *PRDM1*, is a master regulator of plasma cell differentiation (77). *Prdm1^fl/fl^Cd19^Cre^* mice display impaired immunoglobulin secretion and impaired plasma cell formation (78). ~25% ABC-DLBCLs have an inactivating mutation in *PRDM1* but not GCB-DLBCLs (79). Conditional deletion of mouse BLIMP1 in *Prdm1^fl/fl^Cd19^Cre^* displays a significant increase of GC B cells and extrafollicular marginal B cells upon immunization. These mice display splenomegaly and lymphoproliferative disease by 16 months of age (46). Deletion of BLIMP1 in germinal center B cells in *Prdm1^fl/fl^Cγ1^Cre^* mice results in similar expansion of GC upon immunization with comparable splenomegaly and lymphoproliferative disease (46). In both models, mice occasionally develop into DLBCL that are IRF4 positive and negative for differentiation markers, suggesting post-GC origin. These tumors also display nuclear NFκB p50, indicating constitutive active NFκB (46).

BLIMP1 mutation frequently co-occurs with other genetic lesions in driving lymphomagenesis. Introduction of IKK2^ca^ mutation to activate canonical NFκB signaling, cooperates with the GC-conditional loss of function in BLIMP1 to drive progression towards ABC-DLBCL (47). Concurrent NFκB activation and BLIMP1 deletion result in shorter survival and enlarged spleens. Phenotypically, these mice show impaired plasma cell differentiation and reduced serum IgG1 compared with aberrant NFκB activation alone, consistent with BLIMP1 functioning as a tumor suppressor that normally constrains lymphomagenesis (47). Moreover, a compound-genetic model incorporating p53 loss, constitutive NFκB activation, and BLIMP1 dysregulation supports the cooperation of genome instability in driving ABC-DLBCL (48). Tumors from these mice display features of *MYC* upregulation to sustain growth, and immune evasion through reduced MHC class II expression, upregulation of PD-L1, and evidence of T-cell exhaustion (48). These findings provide a rationale for combinatorial therapy for ABC-DLBCL utilizing immune checkpoint blockade and anti-CD20 immunotherapy in further investigation.

### BTG1

(B cell translocation gene 1) missense mutations are found in 70% of MCD cluster DLBCL and are associated with significantly poorer prognosis (3). BTG1 belongs to the BTG/transducer of ERBB2 (TOB) family of anti-proliferative proteins that collectively play a role in regulating cell cycle progression, apoptosis and differentiation (80). BTG1 has been implicated in regulating B cell differentiation across multiple developmental stages and is particularly associated with lymphomas (81). Resequencing of lymphoma patients identifies Q36H as the most frequent BTG1 mutation and confers a conformational change. *R26^lsl.Btg1Q36H/+^ Cd19^Cre/+^* mice carrying the *Btg1^Q36H^* mutation confer a progressive competitive advantage over time in B cells entering the GC following antigen-specific T-dependent immunization (49). This competitive advantage is driven by enhanced activation of biosynthetic pathways, mitochondria function, and most prominently, MYC targets. *Btg1*^Q36H^ affects normal BTG association with the *Myc* gene, and as a result, lowers the threshold for MYC protein synthesis. This endows B cells carrying *Btg1^Q36H^* “super competition” within the GC for proliferation by lowering the threshold required of T cell help and increasing MYC expression to accelerate LZ/DZ cell cycling (49). Nevertheless, *Btg1^Q36H^* alone does not drive mortality in mice even under chronic immunization conditions. Instead, the transfer of haemopoietic stem cells from *R26^lsl.Btg1Q36H/+^Cγ1^Cre/+^* with BCL2 overexpression accelerates DLBCL progression in recipient animals (49). Finally, BTG1 inactivation promotes lymphoma dissemination through the BCAR-RAC1 pathway, suggesting targeting this pathway may be used for lymphomas with loss-of-function BTG1 (82).

### TBL1XR1

Is the key component of the SMRT/NCOR1 complexes and functions as a transcription repressor for balancing the normal GC program (83). Approximately 35% of MCD cluster DLBCL patients harbor *TBL1XR1* mutation, most being heterozygous missense mutations (3). *Tbl1xr1^D370Y^Cγ1^Cre^* mice display impaired GC quantity when immunized with T-dependent antigen, phenocopying the complete loss of Tbl1xr1 in *Tbl1xr1^ko/ko^* animals (50). *Tbl1xr1^D370Y^* causes a significant reduction in GC cell proliferation by interacting with BACH2 at the expense of BCL6, thereby driving the expansion of pre-memory B cell compartment. Upon antigen re-stimulation, *Tbl1xr1^D370Y^* pre-memory B cells preferentially re-enter the GC reaction rather than differentiate into plasma cells (50). Finally, to study lymphoma development, the *VavP-Bcl2* allele is introduced to *Tbl1xr1^ko/ko^Cd19^Cre^*. Histological analysis of these mice reveals large blast B cell lymphomas mimicking the appearance of human ABC-DLBCL (50). This unforeseen malignant transformation proposes a novel cell-of-origin for ABC-DLBCLs and identifies a new target for ABC-DLBCL treatment and may potentially serve as a predictor for patients with extra-nodal lymphoma.

## Perspective and conclusion

**Figure 2:**
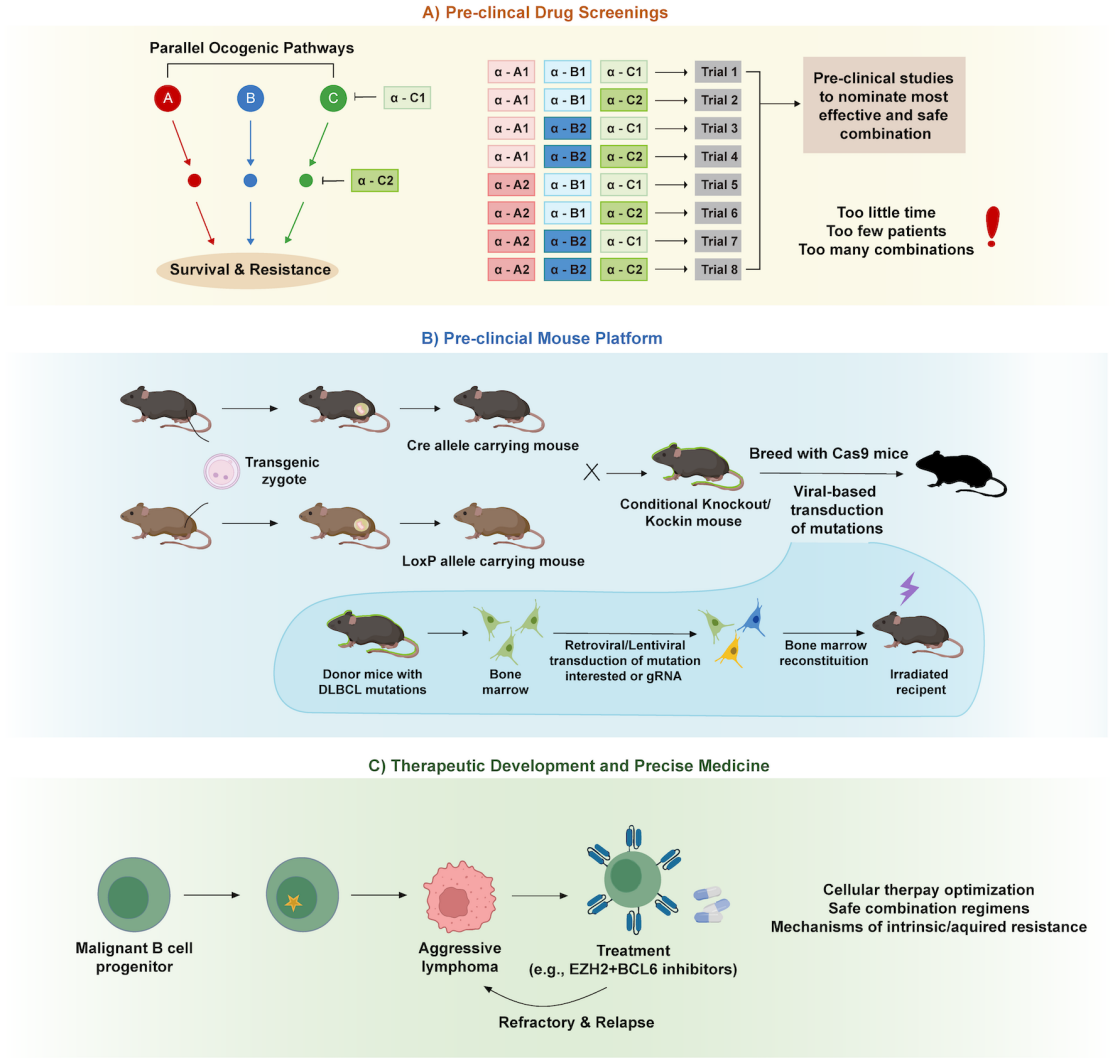
Translating therapeutic discovery with genetic models of DLBCLs. Top panel represents current clinical challenges whereby targeting multiple cooperating oncogenic pathways empirically is limited by factors such as time, patient availability and too many possible combinations. Middle panel shows strategies for using mouse models to faithfully recapitulate genetic events in human lymphomas combining different gene manipulation approaches (i.e. GEMM + CRISPR). Bottom panel displays the potential therapeutic benefits gained from testing drug combinations in genetically engineered B cells in mice by targeting two cooperating pathways (e.g., EZH2 inhibitor + BCL6 inhibitor) in refractory lymphoma.

DLBCL exhibits substantial genetic heterogeneity, with distinct gene mutations that define molecular subtypes, their respective oncogenic mechanisms and therapeutic vulnerabilities. A practical question towards precision medicine in treating lymphoma is confounded by the heterogeneous nature of this disease. Rationalizing treatments should target both the gene mutations that drive lymphoma growth along with overcoming adaptive resistance (**Figure 2**). Empirically, this creates a combination of possibilities that is not feasible to test in clinical settings due to 1) too little time; 2) too few patients; and 3) too many combinations. To overcome this bottleneck, animal models based on known molecular classification of lymphomas that likely share similar affected pathways or genes become an essential tool for advancing the therapeutic treatment of DLBCL. For example, the understanding of how EZH2 mutations cooperate with BCL6 to drive germinal center lymphoma has provided insights into the possible development of combination therapies targeting EZH2 and BCL2 for lymphomas molecularly classified as EZB (**Figure 2**).

However, a key challenge in current genetically engineered mouse models (GEMMs) of lymphoma is that, in most cases, single mutations are not sufficient to fully cause the malignant transformation of a normal B cell (see **Table 1**), requiring approaches that allow us to simultaneously and rapidly introduce new oncogenes and/or delete tumor suppressors on existing genetic background of interest. This can be exemplified by compounding multiple hallmark mutations in a particular lymphoma subtype (45). Alternatively, this can be done by CRISPR-Cas9 approaches to delete tumor suppressors or by retroviral expression of additional oncogenes (45). In this case, this study had demonstrated the requirement of self-reactive BCR and TLR9 for the outgrowth of germinal center B cells in *Myd88^p.L252P^Cd19^Cre^* mice by delivering gRNA targeting either the BCR or TLR9 in a mixed bone marrow chimera approach. The same approach may also be used to model other hallmark mutations in this model, such as deletion of *Prdm1* in order to test its tumor suppressor function. Of note, an advantage of such approach is a fast turnaround time in transduced bone marrow chimera setting of three to six months comparing to traditional gene targeting approach in mice embryo that often require two or more generations of mice breeding (84).

MCD subtype tumors also display high frequency mutations in genes that are involved in interaction with the tumor microenvironment (3). A recent finding suggests that MCD (*Myd88^p.L252P^*) mouse model have altered T cell in the lymphoma niche that both supports tumor growth and influences their therapeutic outcome towards immune checkpoint blockade and CAR-T therapy (85). This finding highlights the need for future work to understand how tumor microenvironment shapes lymphomagenesis of different molecular subtype of DLBCLs and their therapeutic responses. In this regard, the use of GEMMs offers significant advantages compared to patient derived xenografts (PDXs) or cell-line derived xenografts (CDXs). The latter is often cultured for many passaged before implanted into immunocompromised mice, such as NOD-SCID or Nude mice. While this approach is useful, and is being widely adopted, for drug efficacy studies; this type of immunodeficient host marks it insufficient for representing lymphoma biology in human and for translational purposes. PDXs in human CD34+ implanted humanized mice offers a compromise to this approach but is challenged by low success rate owing in part to difficulties with engraftment rate and onset of graft-versus-host disease (86).

Despite a large number of B-cell-specific mouse models being applied to study mutations that directly affect the molecular pathways driving lymphoma, our latest understanding of lymphoma genetics also suggests additional factors such as proteasome dysregulation, antigen presentation, and immune interaction to be also important to consider for modeling lymphomagenesis in animal models and for lymphoma treatment. Future animal models could also include these as novel focuses that may translate into new therapeutic opportunities for lymphoma treatment.
